# Navigating Preclinical Models and Medications for Peripheral Neuropathy: A Review

**DOI:** 10.3390/ph17081010

**Published:** 2024-07-31

**Authors:** Abdulmajeed M. Jali, David Banji, Otilia J. F. Banji, Khalid Y. Hurubi, Faisal Y. Tawhari, Atheer A. Alameer, Atyaf S. Dohal, Raha A. Zanqoti

**Affiliations:** 1 Department of Pharmacology and Toxicology, College of Pharmacy, Jazan University, Jazan 45142, Saudi Arabia; dbanji@jazanu.edu.sa (D.B.); khalidhurubi@gmail.com (K.Y.H.); tfai1sall@gmail.com (F.Y.T.); atheeralameerr@gmail.com (A.A.A.); atuafd@gmail.com (A.S.D.); rzanqoti@gmail.com (R.A.Z.); 2 Department of Clinical Pharmacy, College of Pharmacy, Jazan University, Jazan 45142, Saudi Arabia; obanji@jazanu.edu.sa

**Keywords:** peripheral neuropathy, preclinical models, genetic, non-genetic, drug induced, advantages, disadvantages

## Abstract

Peripheral neuropathy (PN) is a multifaceted disorder characterised by peripheral nerve damage, manifesting in symptoms like pain, weakness, and autonomic dysfunction. This review assesses preclinical models in PN research, evaluating their relevance to human disease and their role in therapeutic development. The Streptozotocin (STZ)-induced diabetic rat model is widely used to simulate diabetic neuropathy but has limitations in faithfully replicating disease onset and progression. Cisplatin-induced PN models are suitable for studying chemotherapy-induced peripheral neuropathy (CIPN) and closely resemble human pathology. However, they may not fully replicate the spectrum of sensory and motor deficits. Paclitaxel-induced models also contribute to understanding CIPN mechanisms and testing neuroprotective agents. Surgical or trauma-induced models offer insights into nerve regeneration and repair strategies. Medications such as gabapentin, pregabalin, duloxetine, and fluoxetine have demonstrated promise in these models, enhancing our understanding of their therapeutic efficacy. Despite progress, developing models that accurately mirror human PN remains imperative due to its complex nature. Continuous refinement and innovative approaches are critical for effective drug discovery. This review underscores the strengths and limitations of current models and advocates for an integrated approach to address the complexities of PN better and optimise treatment outcomes.

## 1. Introduction

Peripheral neuropathy (PN) is a severe medical condition characterised by damage or dysfunction to the peripheral nerves, resulting in a wide range of sensory, motor, and autonomic symptoms. This condition can result from a variety of sources, including metabolic disorders, medication side effects, chemical exposure, and physical trauma. Among these, diabetes mellitus is a noteworthy cause of PN. Given the global increase in diabetes cases, PN, as a complication of diabetes, significantly contributes to both morbidity and mortality rates. Studies indicate that factors such as the type and duration of diabetes, glycaemic control, and co-existing health conditions influence the prevalence of PN. Research conducted by Tesfaye et al. (2010) and Alhajji et al. (2021) [1,2] highlights the multifaceted nature of PN and its complex relationship with diabetes.

**Figure 1:**
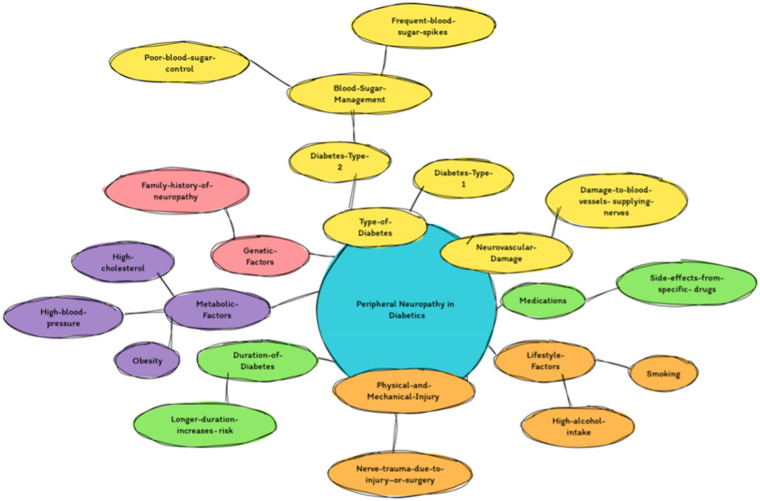
The causes and risk factors of peripheral neuropathy in diabetic patients. It starts with the central node titled “Peripheral Neuropathy in Diabetics”. It branches into several categories. Type of Diabetes: Outlines Diabetes Type 1 and Type 2, highlighting issues related to blood sugar management, including poor control and frequent spikes. Duration of Diabetes: Indicates that a longer duration of diabetes increases the risk of developing neuropathy. Lifestyle Factors: Lists smoking and high alcohol intake as contributory lifestyle risks. Metabolic Factors include high blood pressure, high cholesterol, and obesity. Genetic Factors: Mentions a family history of neuropathy as a genetic risk. Neurovascular Damage: Covers damage to blood vessels that supply nerves. Medications: Side effects from specific drugs that can lead to neuropathy. Physical and Mechanical Injury: Includes nerve trauma due to injury or surgery.

Peripheral neuropathy, while commonly associated with diabetes, can also be induced by specific medications and chemotherapeutic agents, highlighting the multifaceted origins of this significant complication. Among these are antibiotics, antivirals, anticonvulsants, and chemotherapy drugs, which can cause nerve damage and lead to neuropathic symptoms such as pain, numbness, tingling, and weakness [3,4] (Figure 1).

Furthermore, exposure to environmental toxins and industrial chemicals poses a significant risk for peripheral nerve injury. Occupational contact with harmful substances like lead, mercury, organic solvents, and pesticides has been associated with the onset of peripheral neuropathy among workers in various industries [5,6].

**Figure 2:**
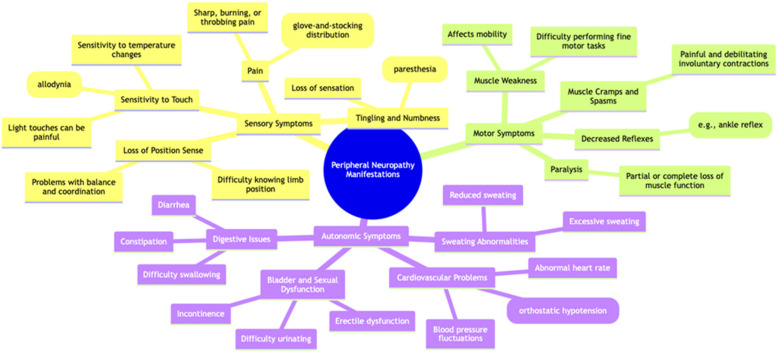
Peripheral neuropathy manifestations.

The clinical presentation of PN can vary significantly based on the specific nerves affected and the underlying cause. Sensory symptoms, such as burning pain, tingling, and numbness, are frequently observed, while motor symptoms may include weakness, muscle atrophy, and difficulty with coordination. Autonomic dysfunction can also present as changes in blood pressure, heart rate, sweating, and gastrointestinal function [7] (Figure 2).

Peripheral neuropathy presents a significant public health issue, with extensive ramifications for healthcare utilization, economic burden, and individual quality of life. Identifying its multifaceted and diverse causes and its comprehensive range of clinical presentations is crucial for creating effective prevention, diagnostic, and treatment methodologies.

The management of peripheral neuropathy centers on managing the underlying condition causing the neuropathy and alleviating symptoms. Commonly prescribed medications include anticonvulsants, antidepressants, opioids, topical therapies, and other skin preparations. Gabapentin and pregabalin are anticonvulsants prescribed to mitigate the painful symptoms of peripheral neuropathy by modulating hyperactive neurons that transmit pain signals to the brain [8]. Some antidepressant medications can affect the brain and spinal cord’s chemical processes, which may result in pain relief. Tricyclic antidepressants, such as amitriptyline, doxepin, and nortriptyline, are effective in treating neuropathic pain. Additionally, serotonin and norepinephrine reuptake inhibitors (SNRIs), such as duloxetine and venlafaxine, have demonstrated effectiveness in managing pain associated with diabetic neuropathy. Opioids are generally reserved for severe pain resistant to other treatments due to their potential for addiction and other adverse effects. Medications such as tramadol or oxycodone may be administered under controlled and monitored conditions [9,10].

Moreover, topical therapies, medications, or substances applied to the skin or mucous membranes to produce a local effect, such as capsaicin cream, which comprises a substance extracted from hot peppers, can modestly alleviate the intensity of pain signals transmitted from the skin to the brain. Similarly, a lidocaine patch, which functions as a local anesthetic, can also mitigate pain by desensitizing the area where it is applied.

Additionally, topical agents delivered via skin patches, gels, creams, or sprays can assist in alleviating pain. These may contain botanicals or compounds that act locally rather than affecting the entire body. Patients must collaborate closely with their healthcare provider to devise a personalized treatment plan that provides the optimal balance between symptom relief and minimizing side effects. Each of these therapies has potential side effects, and their efficacy can fluctuate depending on the individual and the root cause of the neuropathy [10].

In today’s medical landscape, the urgent need for effective treatments for PN is evident. With the increasing incidence of chemotherapy-induced neuropathy, there’s a critical demand for improved management strategies. Furthermore, using sophisticated experimental preclinical models proves crucial in expediting the drug discovery process for PN. These models enable scientists to simulate and examine the intricate mechanisms associated with PN development and progression in a regulated laboratory environment. Through applying such models, researchers can acquire substantial knowledge about PN pathophysiology and evaluate innovative therapeutic strategies’ effectiveness and safety.

The study of PN and the development of novel therapies to address it is of utmost importance in modern healthcare. The progress made in this field has the potential to significantly improve the quality of life for patients with PN and open up new avenues for effective treatment in the future. Consequently, ongoing research efforts to elucidate and mitigate PN and its associated side effects remain highly pertinent and influential in contemporary medical research.

The central objective of this study is to explore the various preclinical models utilized in studying peripheral neuropathy (PN), a pressing medical concern. The goal is to provide valuable insights to scientists and medical professionals regarding the advantages and disadvantages of these models, thereby assisting them in selecting the most appropriate model for their specific research requirements. Additionally, this analysis will include the introduction of medications such as gabapentin, pregabalin, duloxetine, and fluoxetine, which have emerged as potential treatments for PN. This study aims to enhance understanding of their efficacy and relevance in PN research by showcasing the results of these medications in the reviewed models.

## 2. Peripheral *Neuropathy*: Preclinical *Models*

### 2.1. Disease-Induced Model

Preclinical research frequently employs animal models to investigate diseases and evaluate potential treatments. Rodent models, which share numerous physiological similarities with humans, are often selected due to their accessibility and ease of manipulation.

The use of a diabetic rat model induced by streptozotocin (STZ) is a common practice. STZ, derived from Streptomyces bacteria, is administered to adult rats to induce diabetes by explicitly targeting and damaging the insulin-producing β cells in the pancreas. This model effectively simulates type 1 diabetes in humans and is widely utilised for studying diabetic neuropathy and evaluating therapeutic interventions [11].

Another model utilizes genetically modified rodents that exhibit spontaneous diabetes. These animals acquire diabetes due to genetic mutations that impact insulin production or signalling pathways. The use of spontaneous diabetic models enables researchers to investigate the natural progression of diabetes and its associated complications over extended periods [12].

In addition, some diet-induced models involve feeding rodents high-fat or high-sugar diets to induce obesity and insulin resistance, which mimic certain aspects of type 2 diabetes. These models are valuable for studying metabolic syndrome and its associated complications, such as neuropathy, as demonstrated by Surwit and Kuhn (1988) [13]. The model selection depends on the study’s particular research inquiries and objectives, as each model has unique advantages and restrictions.

### 2.2. Streptozotocin (STZ)-Induced Diabetic Rat Model

The use of the STZ-induced diabetic rat model is prevalent in diabetes research for investigating the pathophysiology of the disease and its complications, such as diabetic neuropathy. STZ, a toxin derived from Streptomyces bacteria, targets explicitly and damages insulin-producing β cells in the pancreas, resulting in insulin deficiency and hyperglycaemia, which simulates type 1 diabetes in humans [12].

This model is beneficial for exploring numerous facets of diabetic neuropathy, including the underlying mechanisms, neuropathic pain, and promising therapeutic interventions. Investigators may employ diverse dosing regimens, such as administering a single high dose or multiple lower doses over an extended period [11], to induce diabetes in rats for experimental purposes.

The STZ-induced diabetic rat model is an adequate representation of human disease and serves as a platform for investigating potential interventions to alleviate neuropathic symptoms and hinder disease progression.

Streptozotocin (STZ)-induced diabetes in mice is a widely used model to study the pathophysiological mechanisms of DN. Beyond the direct effects of diabetes, DN significantly impacts organs innervated by the affected nerves. One of the critical tissues influenced by DN is skeletal muscle.

In STZ-induced diabetic mice, skeletal muscles undergo significant alterations due to the metabolic disturbances of diabetes and the secondary effects of DN. Diabetes induces a shift in muscle fibre types, changes in fibre diameter, and an increase in the relative capillarization of skeletal muscle. These changes result from the direct hyperglycemic environment and the indirect effects mediated by neuropathic alterations [14].

The direct impact of hyperglycaemia on skeletal muscle includes the dysregulation of glucose and lipid metabolism, leading to insulin resistance, muscle atrophy, and altered muscle fibre composition. Specifically, there is a shift from oxidative type I fibres to more glycolytic type II fibres, which affects muscle function and endurance [15].

Indirectly, DN exacerbates these changes by impairing the neural input to the muscles. This neural impairment leads to muscle weakness, reduced coordination, and further atrophy. The compromised neural stimulation affects the muscle fibres’ trophic support, contributing to muscle fibre diameter alterations and increased capillarization as a compensatory mechanism to maintain oxygen and nutrient delivery [16].

Therefore, understanding the comprehensive impact of diabetes and DN on skeletal muscles is crucial for developing therapeutic strategies to mitigate these changes and improve the quality of life for individuals with diabetes.

#### 2.2.1. Advantages

The STZ-induced diabetic rat model is a valuable instrument for investigating diabetic neuropathy, as it reproduces crucial functional and biochemical irregularities observed in humans [17,18]. This economical and extensively utilized model provides valuable insights into multiple facets of neuropathy progression.

In addition to hyperglycemia, the STZ-induced diabetic rat displays significant deficits in nerve conduction velocities, calcium signalling, impairments in mitochondrial function, and activation of apoptotic pathways within four weeks of diabetes onset [19]. These early changes correspond to those observed in human diabetic neuropathy, providing researchers with a reliable platform to explore the underlying mechanisms and test potential therapeutic interventions.

Overall, the STZ-induced diabetic rat model effectively reflects critical aspects of nerve dysfunction in human diabetic neuropathy. It is a valuable tool for advancing our understanding of this debilitating complication and developing novel treatment strategies [18].

#### 2.2.2. Disadvantages

The STZ diabetic rat model is employed in studies related to diabetes research; however, it presents various challenges that researchers must confront to guarantee the dependability and consistency of their outcomes. One significant issue is the inconsistent induction of hyperglycemia, which can arise due to disparities in the efficiency of the low-dose STZ method. Not all injected rats may develop diabetes, indicating potential technical problems that must be addressed to achieve reliable results.

Moreover, discrepancies in outcomes are another concern associated with this model. Variations in the potency of STZ across batches can lead to disparities in the severity of diabetes between and within laboratories. This inconsistency impacts several critical aspects, such as the proportion of rats developing diabetes, the extent of insulin deficiency, the onset and magnitude of neuropathic changes, and even survival duration.

The STZ model has been utilized across different species, encompassing mice, rats, and larger animals such as dogs and pigs. The selection of animal models can profoundly affect study outcomes owing to variations in physiology and metabolism. While mice and rats are frequently chosen for their convenient size and well-understood genetics, they may not completely mirror the pathology of diabetic neuropathy in humans [20].

STZ is administered as a single high dose or multiple lower doses to induce diabetes. The single high-dose method is straightforward and induces rapid onset of diabetes but can result in significant variability in glucose levels and higher mortality rates. In contrast, multiple low-dose STZ injections can produce a more gradual onset of diabetes with more stable hyperglycemia but require more time and careful monitoring. The choice of regimen impacts the study’s reproducibility and animal welfare [21].

The dose of STZ and the time intervals between doses also play a critical role in the model’s effectiveness and reproducibility. Higher doses tend to induce more severe diabetes but at the cost of increased animal morbidity and mortality. Conversely, lower doses may result in less severe diabetes, requiring more extended experimental periods to observe significant neuropathic changes. The variability in dosing regimens across studies can complicate comparing results and interpretations [22].

One significant disadvantage of STZ models is the high morbidity and mortality rates associated with the induction of diabetes. Animals often experience substantial weight loss, reduced mobility, and other health issues, necessitating close monitoring and care. High morbidity rates can lead to ethical concerns and increase the complexity of the experiments [23].

Due to the high morbidity and mortality rates, maintaining a large cohort of diabetic animals can be costly. The need for specialized care, frequent monitoring, and potentially high attrition rates contribute to the cost of STZ model studies. Additionally, the variability in outcomes requires larger sample sizes to achieve statistically significant results, increasing costs [24].

To overcome these challenges, researchers must meticulously design their experiments and include all necessary experimental groups within a single study. Comparisons across separate studies, even within the same laboratory, can be complicated due to the variability in STZ potency and the resulting differences in diabetes severity among rat cohorts.

In conclusion, the STZ diabetic neuropathy model presents difficulties interpreting and contrasting results due to the variability in STZ potency and the resulting diabetes severity in rats. To ensure the dependability and reproducibility of their findings, researchers must carefully address these challenges [25,26].

#### 2.2.3. Procedures

**Figure 3:**
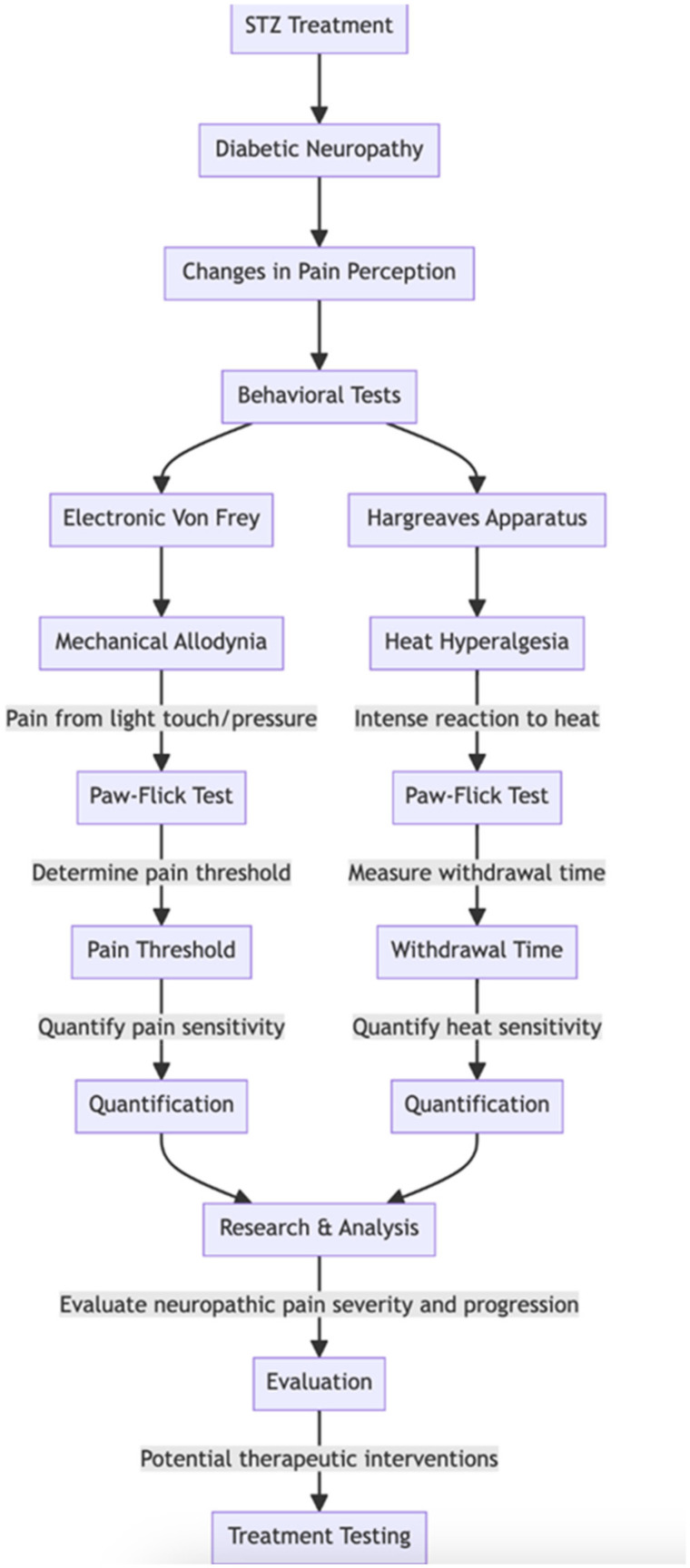
Diagram illustrating the process and significance of behavioural tests for pain perception in STZ-treated rats.

**Figure 4:**
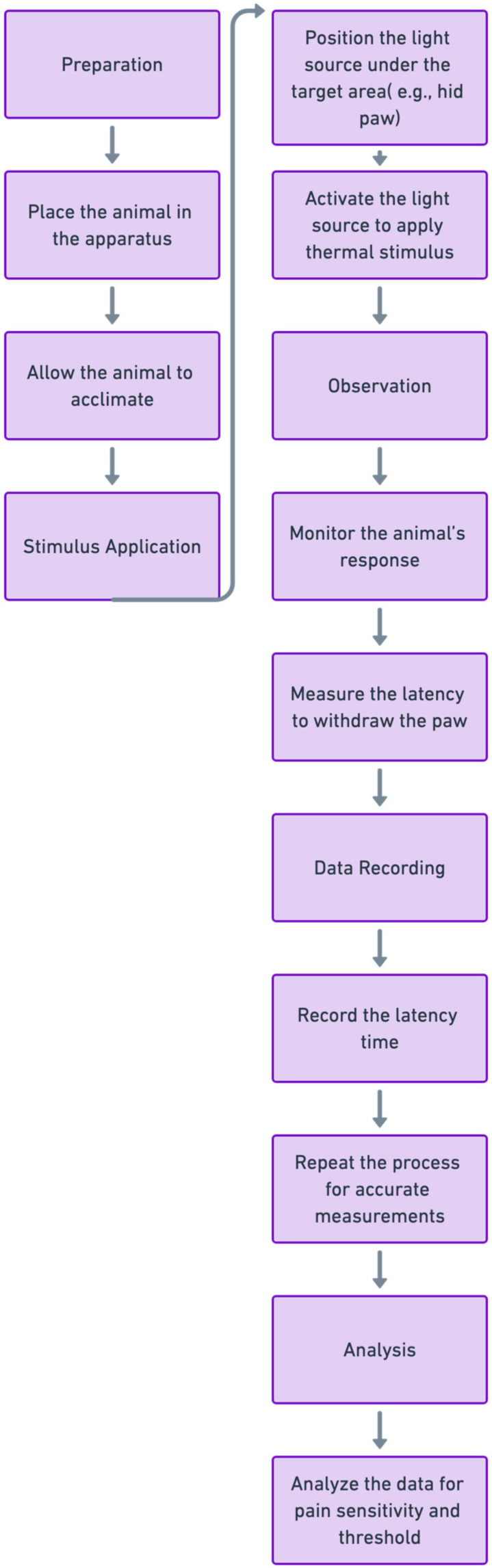
Diagram illustrating the process of using the Hargreaves Apparatus, also known as the Paw-Flick Test, to assess heat hyperalgesia in rodents. The flowchart begins with placing the rodent in the apparatus and proceeds through the steps of the test, including focusing the radiant heat on the rodent’s paw, triggering the withdrawal reflex, measuring the latency period, and recording the withdrawal latency. The results are then interpreted, with shorter withdrawal latencies indicating increased sensitivity to heat, which is a sign of hyperalgesia. The process ends after the interpretation of results, determining the presence and extent of hyperalgesia in the test subject.

Rats with STZ-induced diabetic neuropathy exhibit changes in pain perception, presenting a valuable opportunity for researchers to assess these changes using behavioral tests quantitatively. Among the various methods available, two commonly used assays in the STZ diabetic neuropathy model are the electronic Von Frey and Hargreaves apparatus, which measure mechanical allodynia and heat hyperalgesia through the Paw-Flick Test (Figure 3). Mechanical allodynia refers to the perception of pain in response to non-painful stimuli, such as light touch or pressure. The electronic Von Frey apparatus applies controlled mechanical pressure to the rat’s paw, allowing researchers to determine the threshold at which the animal perceives discomfort. On the other hand, heat hyperalgesia involves an exaggerated response to painful stimuli, specifically heat. The Hargreaves apparatus measures the time it takes for the rat to withdraw its paw from a radiant heat source, indicating heightened sensitivity to heat (Figure 4) [27].

These behavioral tests allow researchers to quantitatively evaluate the abnormal pain sensations that develop as complications of diabetic neuropathy in STZ-treated rats. By systematically assessing mechanical allodynia and heat hyperalgesia, researchers can gain valuable insights into the progression and severity of neuropathic pain in this model. This quantitative approach enhances the objectivity and reproducibility of pain assessment, facilitating the evaluation of potential therapeutic interventions to alleviate neuropathic pain in diabetic neuropathy [27].

### 2.3. Detection of Diabetic Neuropathies in Studies

The detection of diabetic neuropathy in murine models typically involves a combination of behavioral, electrophysiological, and histological tests, along with advanced imaging techniques. Below are the expanded methods used to detect neuropathy in murine models.

Behavioral Tests: Behavioral tests are commonly employed to assess sensory deficits in diabetic neuropathy. One of the most widely used tests is the von Frey filament test for mechanical allodynia. This test involves applying a series of calibrated filaments to the mouse’s paw to decide the threshold at which the animal responds to a mechanical stimulus, indicating pain sensitivity [25].

Electrophysiological Tests: Electrophysiological tests provide quantitative data on nerve function. These tests measure parameters such as nerve conduction velocity and amplitude, which can indicate neuropathic changes. Electrophysiological assessments are crucial for understanding the extent of nerve damage and dysfunction in diabetic neuropathy [28].

Histological Analyses: Histological analyses involve examining the physical structure of nerves to assess neuropathic changes. One important method is quantifying nerve fibre density, which can reveal the extent of nerve degeneration and loss. This type of analysis provides insights into the structural damage caused by diabetic neuropathy [29].

Radiological Modalities: One crucial aspect often neglected is the detection of neuropathies using radiological modalities such as diffusion tensor imaging (DTI). DTI enables the depiction of nerve fascicles and can provide detailed insights into the integrity and pathology of nerves and fascicles. This imaging technique can be applied both ex vivo and in vivo, offering a non-invasive method to study nerve structure and function [30].

By combining these methods, researchers can comprehensively understand the extent and nature of diabetic neuropathy in murine models. This multifaceted approach allows for more accurate detection and characterization of neuropathic changes, ultimately aiding in developing effective therapeutic strategies.

### 2.4. Assessment of Mechanical Allodynia: The Electric Von Frey Method

**Figure 5:**
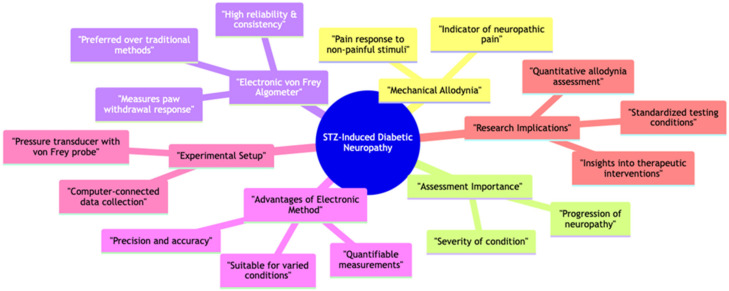
This mind map provides a structured overview of the critical components of studying diabetic neuropathy induced by streptozotocin (STZ) in rats. The model focuses on the assessment of mechanical allodynia. The central idea revolves around the advantages of using an electronic von Frey algometer over traditional methods, emphasizing its precision, accuracy, and ability to provide quantifiable measurements. The mind map breaks down the importance of assessing mechanical allodynia, the benefits of the electronic method, the experimental setup involving a pressure transducer and computerized data collection, and the implications for research, such as standardized testing conditions and valuable insights into potential therapeutic interventions.

In animals with diabetic neuropathy induced by streptozotocin (STZ), mechanical allodynia serves as a crucial indicator of neuropathic pain development. Assessing mechanical allodynia is essential in understanding the progression and severity of diabetic neuropathy. While traditional methods using standard Von Frey hairs are employed, electronic von Frey algometers have emerged as the preferred choice due to their higher reliability and consistency, particularly in varied environmental conditions (Figure 5).

The electronic von Frey algometer presents several advantages over traditional methods. Its precision and accuracy are enhanced, ensuring more reliable measurements. Furthermore, the device allows for the quantification of mechanical sensitivity both before and after diabetes induction. This is achieved by measuring the paw withdrawal response to innocuous mechanical stimuli and quantitatively assessing the animal’s sensitivity to touch.

The setup typically comprises a handheld pressure transducer connected to a computer via a von Frey probe for data collection and display. This configuration enables researchers to precisely control the applied force and rate of stimulus application, ensuring standardized testing conditions across experiments. By recording these parameters, researchers can accurately quantify mechanical allodynia in STZ diabetic rats, providing valuable insights into the effectiveness of therapeutic interventions and disease progression [31].

### 2.5. Assessment of Heat Hyperalgesia: Hargreaves Apparatus (Paw-Flick Test)

**Figure 6:**
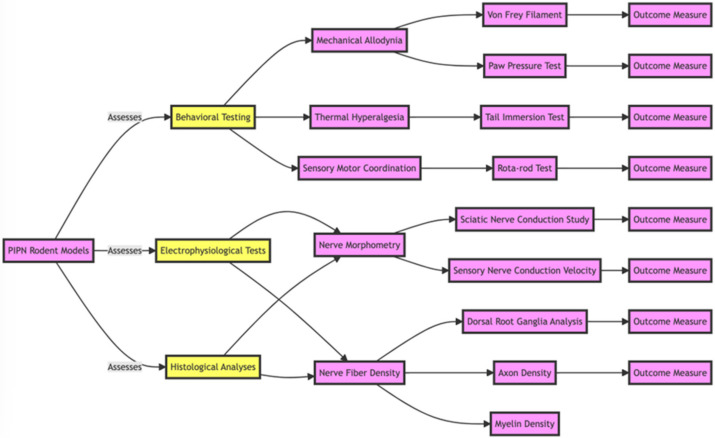
Diagram illustrating the various outcome measures used to evaluate neuropathy in rodent models of PIPN.

Evaluating heat hyperalgesia, or heightened sensitivity to painful stimuli, is critical in clinical and research settings for understanding pathological conditions, such as neuropathic pain or inflammatory responses. One widely employed method for assessing heat hyperalgesia in rodents is the Hargreaves Apparatus, often called the Paw-Flick Test. Michael Hargreaves developed this apparatus in 1988 to measure thermal nociception in rodents. This test employs a radiant heat source focusing on the animal’s paw, triggering a withdrawal reflex. The latency period between applying the heat stimulus and the withdrawal response is then measured. Shorter withdrawal latencies indicate increased sensitivity to heat, suggesting hyperalgesia. In a study by Hargreaves et al. (1988) [32], the authors described the apparatus and its application in assessing thermal nociception in rats. They found that the withdrawal latency was sensitive to alterations in skin temperature and provided a reliable measure of thermal nociception (Figure 6).

Moreover, Deuis et al. (2017) emphasized the pivotal role of the Hargreaves Apparatus in preclinical pain research [33]. This test demonstrates a remarkable ability to differentiate between diverse analgesic agents and is highly reliable in evaluating heat hyperalgesia in animal models. The Hargreaves Apparatus extensively studied pain mechanisms and evaluated potential analgesic compounds. For example, a study by Mao et al. (2002) [34] employed this method to investigate the analgesic effects of cannabinoid receptor agonists in a rat model of neuropathic pain.

### 2.6. Collective Studies

The effects of gabapentin, pregabalin, and duloxetine on the STZ-induced diabetic peripheral neuropathy (DPN) model have been thoroughly examined, and meticulous documentation of their outcomes has been conducted (Table 1).

#### 2.6.1. Gabapentin

Gabapentin is a medication primarily utilized to treat neuropathic pain, particularly in individuals with diabetic neuropathy. In animal models of STZ-induced DPN, gabapentin has exhibited its potential to alleviate neuropathic pain symptoms, such as mechanical allodynia and thermal hyperalgesia. The study conducted by Hamidi et al. (2014) revealed that gabapentin treatment resulted in a significant reduction in mechanical allodynia in rats with STZ-induced DPN [35]. The researchers discovered that gabapentin effectively reversed the heightened sensitivity to mechanical stimuli commonly found in individuals with diabetic neuropathy [36]. Another study by Kilic et al. (2012) investigated the analgesic effects of gabapentin in STZ-induced DPN mice [37]. The findings demonstrated that gabapentin administration effectively diminished thermal hyperalgesia, indicating its effectiveness in reducing pain sensitivity associated with diabetic neuropathy.

#### 2.6.2. Pregabalin

Pregabalin, like gabapentin, serves as an anticonvulsant medication employed in the treatment of neuropathic pain conditions, including diabetic neuropathy. Research by Verma et al. (2014) has demonstrated the effectiveness of pregabalin in mitigating mechanical allodynia and thermal hyperalgesia in STZ-induced DPN models [38]. Additionally, a study by Demir et al. (2021) revealed the neuroprotective potential of pregabalin in STZ-induced DPN rats, as it alleviated neuropathic pain symptoms and decreased oxidative stress markers in diabetic rats [39].

#### 2.6.3. Duloxetine

Duloxetine is a commonly prescribed serotonin–norepinephrine reuptake inhibitor (SNRI) for the management of neuropathic pain resulting from diabetic neuropathy. Numerous studies have shown that duloxetine possesses analgesic effects and improves sensory function in STZ-induced DPN models. For instance, a study conducted by Calcutt et al. (2008) investigated the impact of duloxetine on mechanical allodynia and thermal hyperalgesia in STZ-induced DPN rats [40]. The results indicated that duloxetine treatment significantly reduced both mechanical allodynia and thermal hyperalgesia, suggesting its potential to alleviate neuropathic pain symptoms associated with diabetic neuropathy. Similarly, a study by Mixcoatl-Zecuatl and Jolivalt (2011) explored the analgesic mechanisms of duloxetine in STZ-induced DPN rats. The findings showed that duloxetine administration attenuated pain behavior and normalized the expression of pain-related proteins in the spinal cord, indicating its ability to modulate central sensitization processes in diabetic neuropathy [41].

Overall, these studies collectively indicate the therapeutic potential of gabapentin, pregabalin, and duloxetine in mitigating pain symptoms and improving sensory function in STZ-induced diabetic peripheral neuropathy models.

#### 2.6.4. Behavioural Tests Used in Neuropathy Models

Von Frey Test: The Von Frey test is a widely used method for assessing mechanical allodynia, where light touch or pressure that would not usually cause pain becomes painful. This test involves using calibrated filaments to apply a specific force to the animal’s paw and measuring the withdrawal response. It is commonly employed in various neuropathy models, including the following.

**Figure 7:**
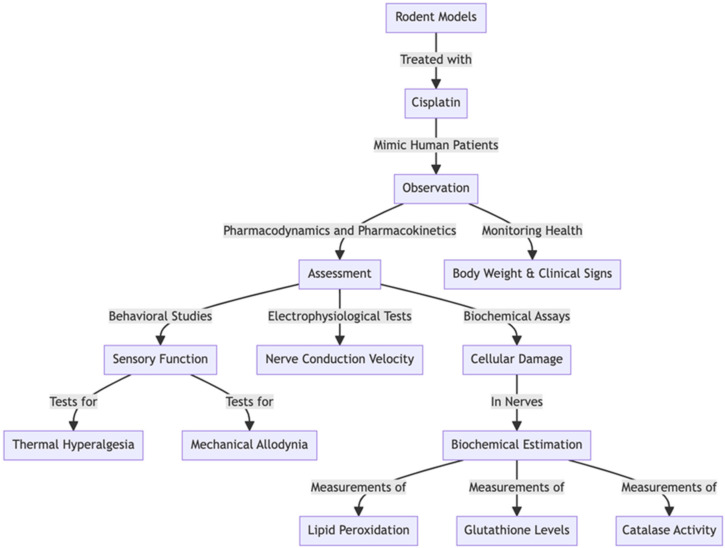
Diagram illustrating the testing procedures for cisplatin-induced peripheral neuropathy models.

Cisplatin-Induced Peripheral Neuropathy (CIPN): In CIPN models, the Von Frey test helps evaluate the development and severity of mechanical allodynia induced by chemotherapy drugs like cisplatin. It is a crucial tool for assessing the efficacy of potential therapeutic agents aimed at reducing neuropathic pain (Figure 7) [42].

STZ-Induced Diabetic Neuropathy: In the STZ-induced diabetic neuropathy model, the Von Frey test measures the progression of diabetic neuropathy and the effectiveness of treatments like gabapentin and pregabalin in alleviating pain [43].

Surgically Induced Model of PN: This test is also applied in surgically induced models of peripheral neuropathy, such as the chronic constriction injury (CCI) model, to assess mechanical allodynia resulting from nerve injury [44].

Paclitaxel-Induced Peripheral Neuropathy: The Von Frey test is utilized in paclitaxel-induced models to study the mechanical pain sensitivity caused by this chemotherapeutic agent. It helps evaluate the protective effects of various treatments against chemotherapy-induced neuropathy [45].

Plantar Test: The Plantar test, also known as the Hargreaves test, measures heat hyperalgesia by assessing the withdrawal latency of the animal’s paw from a heat source [46]. This test is critical for evaluating thermal pain sensitivity in the following different neuropathy models.

Cisplatin-Induced Peripheral Neuropathy (CIPN): In CIPN models, the Plantar test helps understand the thermal pain thresholds and the impact of potential treatments in reducing chemotherapy-induced heat hyperalgesia [47].

STZ-Induced Diabetic Neuropathy: This test assesses heat hyperalgesia in diabetic neuropathy models induced by streptozotocin. It is instrumental in studying the effectiveness of drugs like duloxetine in managing diabetic neuropathic pain [48].

Surgically Induced Model of PN: The Plantar test is applied in surgically induced neuropathy models to evaluate the development of thermal hyperalgesia following nerve injury and to test the efficacy of neuroprotective agents [49].

Paclitaxel-Induced Peripheral Neuropathy: In models of paclitaxel-induced neuropathy, the Plantar test measures heat sensitivity and helps assess the effectiveness of therapeutic strategies to alleviate chemotherapy-induced pain [50].

**Table 1:** A summary of the medications used in the STZ diabetic model.

Study	Medication	Dose	Model Used	Tests Used	Main Findings
Archana and Annnapurna, 2016 [51]	Pregabalin	50 mg/kg	STZ-induced type i diabetic mice and type ii diabetic db/db mice	Paw withdrawal test (PWT)	Circadian rhythms play a significant role in the analgesic properties of PGN in diabetic mice regarding small intestinal uptake. The absorption of PGN is facilitated by the organic cation transporter novel type 1 (Octn1) in the small intestine. The analgesic effect of PGN was heightened during the times of day when Octn1 expression peaked, which enhanced PGN absorption. In short, fluctuating levels of Octn1 in the small intestine of diabetic mice at different times of the day underpinned the time-dependent effects of PGN analgesia.
Kuhad et al., 2008 [52]	Duloxetine	10 and 20 mg/kg	STZ-induced diabetic mouse model	Tail immersion and hot-plate assays	Duloxetine administration to diabetic mice at varying doses reduced dose-dependent pain sensitivity in tail immersion and hot plate tests. Furthermore, duloxetine administration caused dose-dependent increases in neuromodulator adenosine levels in mice. Higher doses of duloxetine provided increasingly significant pain relief and elevated adenosine levels, implying the involvement of the adenosinergic system in the anti-nociceptive mechanism of duloxetine.
Tembhurne et al., 2011 [53]	Fluoxetine	20 mg/kg	Experimental model of diabetes-induced neuropathic pain perception in rat	Blood glucose level test, grip strength test, hot plate test, tail flick test	The administration of fluoxetine significantly reduced blood glucose levels in diabetic animals, demonstrating its hypoglycemic effect. In addition to lowering hyperglycaemia, fluoxetine also seemed to protect against peripheral neuropathy, a common complication of diabetes. Diabetic rats treated with fluoxetine exhibited improved grip strength, suggesting less nerve damage than untreated controls. Fluoxetine also increased paw licking time and withdrawal latency to thermal stimuli, which suggests preserved pain perception and prevention of sensory neuropathy.
Lopez-Soldado, 2003 [26]	Fluoxetine	5, 10, and 20 mg/kg	STZ-induced diabetic mouse model	Tail-immersion and hot-plate assays	The results of a study demonstrated that mice with diabetes exhibited pain-relieving effects when treated with fluoxetine at 10 and 20 mg/kg doses. These effects were heightened by pindolol, an antagonist at 5-HT1A/1B receptors, but reduced by ritanserin, an antagonist at 5-HT2A/2C receptors. Interestingly, ondansetron, a selective 5-HT3 receptor antagonist, did not appear to impact the antinociceptive effects of fluoxetine. The data gathered from the study suggest that fluoxetine’s antinociceptive properties in diabetic mice are mediated by 5-HT1A/1B and 5-HT2A/2C receptors but not 5-HT3 receptors.
Mixcoatl-Zecuatl et al., 2011 [41]	Duloxetine	s.c. 50 mL Systemic administration via i.p. 2 mL/kg Intrathecal (i.t.) 10 mL	STZ-induced diabetic rat model	Tactile allodynia test	Duloxetine displayed effective pain relief by reducing tactile allodynia in diabetic rats. The anti-allodynic impact of duloxetine was diminished by ketanserin or pruvanserin, which suggests that spinal 5-HT2A receptors play a role in the action of duloxetine. In conclusion, the results imply that 5-HT2A receptors in the spinal cord contribute to the anti-allodynic effects of duloxetine in diabetic neuropathic pain.
Hamidi et al., 2014 [35]	Gabapentin	Systemic use: 75 mg/kg Topical use: 10% gel	STZ-induced diabetic neuropathic pain model	Static and dynamic mechanical allodynia and vulvodynia tests	Gabapentin, through systemic or topical means, led to a notable increase in paw withdrawal thresholds for static stimuli and paw withdrawal latencies for dynamic stimuli, as compared to diabetic controls, demonstrating its anti-allodynic properties. Additionally, gabapentin showed a significant improvement in diabetes-associated vulvodynia. To conclude, gabapentin administration, whether central or peripheral, effectively alleviated various types of neuropathic pain and vulvodynia in a rodent model of STZ-induced diabetic neuropathy.
Surcheva et al., 2017 [54]	Gabapentin	60 mg/kg	CCI and STZ-induced diabetes in rats	Evaluation of mechanical, tactile, and heat hypersensitivity	The use of gabapentin has been shown to reduce heightened sensitivity to mechanical, tactile, and heat stimuli in rat models of neuropathic pain.
	Pregabalin	30 mg/kg	CCI and STZ-induced diabetes in rats	Evaluation of mechanical, tactile, and heat hypersensitivity	Pregabalin reduced sensitivity to mechanical, tactile, and heat stimuli in rat models of neuropathic pain.

STZ—streptozotocin, PWT—Paw Withdrawal Test, PGN—pregabalin, Octn1—organic cation transporter novel type 1, 5-HT1A/1B—5-hydroxytryptamine receptor 1A/1B, HT2A/2C—5-hydroxytryptamine receptor 2A/2C, 5-HT3—5-hydroxytryptamine receptor 3, HT2A—5-hydroxytryptamine receptor 2A, CCI—chronic constriction injury.

## 3. Cisplatin-Induced Peripheral Neuropathy

Cisplatin-induced peripheral neuropathy (CIPN) is a frequent and significant adverse effect experienced by patients undergoing treatment with cisplatin, a chemotherapy medication extensively used for the treatment of various cancers, such as ovarian, testicular, bladder, and lung cancers. This neuropathy is a result of the neurotoxic effects of cisplatin on peripheral nerves, which manifest through multiple mechanisms. Cisplatin can accumulate in dorsal root ganglion neurons, leading to damage and cell death, and it increases the production of reactive oxygen species, causing oxidative stress that harms neuronal cells. Additionally, it disrupts mitochondrial function, essential for energy production in nerve cells, and induces inflammatory responses that worsen nerve damage. Symptoms of CIPN include sharp or burning pain, numbness, and tingling, often described as feeling like an invisible glove or sock, increased sensitivity to cold temperatures, and, in severe cases, motor weakness. Effective management of CIPN involves reducing the cisplatin dosage or altering the treatment regimen, using medications such as anticonvulsants (e.g., gabapentin, pregabalin) and antidepressants (e.g., duloxetine) to manage pain and discomfort, and engaging in physical therapy to support mobility and functioning of the affected areas. The onset of CIPN can occur during or shortly after the initiation of treatment, and its severity is often dose dependent. In some cases, symptoms may persist long after the cessation of treatment, significantly affecting the quality of life, making effective management and early intervention crucial for improving patient outcomes and minimizing long-term complications [55].

The relationship between CIPN and DPN is worthy of exploration and evaluation due to their shared pathophysiological mechanisms, treatment responses, and research outcomes. This shared basis offers the potential for informative insights to advance the understanding and treatment of both conditions. The following aspects highlight the connections between CIPN and DPN and the benefits of mutual informativeness [42]. Common Pathological Mechanisms: Both CIPN and DPN exhibit similar pathophysiological mechanisms, such as oxidative stress, mitochondrial dysfunction, neuroinflammation, and altered neuronal ion channel activities. Comprehending these shared mechanisms can help the development of drugs that target these pathways, potentially benefiting patients with either condition. For example, research delineating the role of oxidative stress in CIPN-induced nerve damage could provide insights into antioxidant therapies that may also alleviate similar damage in DPN [56]. Therapeutic Insights: Pharmacological interventions for one condition often offer beneficial insights for the other due to their shared underlying mechanisms. For instance, medications that are effective in mitigating pain and other neuropathic symptoms in CIPN may also be explored for DPN, often resulting in promising outcomes.

An example is pregabalin, a medication commonly used to treat neuropathic pain in CIPN, which is also prescribed for pain management in DPN. The successful application of pregabalin in both conditions highlights the potential for cross-applicability of neuropathic pain treatments [57]. The findings from clinical trials focused on novel therapies for CIPN, such as improving dosages and scheduling, may apply to trials investigating DPN. This could potentially lead to improved patient outcomes. For instance, a clinical trial examining the dosing and timing of a neuroprotective agent for CIPN may provide helpful information that could influence studies on DPN, particularly regarding minimizing side effects and maximizing therapeutic effects [58]. Biomarker Development: Investigating biomarkers for early detection, disease progression, and treatment response in one condition may also hasten similar advancements in the other. Finding biomarkers that effectively predict and monitor chemotherapy-induced peripheral neuropathy could also apply to DPN. For instance, a biomarker showing nerve fibre degeneration in chemotherapy-induced peripheral neuropathy may also be relevant for detecting nerve damage in DPN early, allowing for earlier intervention and potentially improved outcomes [59]. The findings obtained from these models about the development of neuropathy and treatment response can provide valuable information for interventions in both conditions. For example, using an animal model that proves effective nerve regeneration following a particular treatment for CIPN, researchers can test comparable DPN approaches while simultaneously considering the unique diabetic conditions [60]. Utilizing the parallels between chemotherapy-induced peripheral neuropathy and DPN, medical professionals and researchers can augment their comprehension of peripheral neuropathies and devise more effective therapies, thereby improving the quality of life for individuals grappling with these disabling disorders.

### 3.1. Cisplatin-Induced Peripheral Neuropathy Preclinical Model

Cisplatin-induced peripheral neuropathy (CIPN) is a widely accepted preclinical model used to investigate chemotherapy-induced nerve damage. Cisplatin, an often-utilized chemotherapeutic medication, causes peripheral neuropathy characterized by sensory and motor deficits. This model involves administering cisplatin to experimental animals, typically rodents, to replicate the neuropathic effects observed in human patients undergoing chemotherapy.

Numerous studies have employed the CIPN preclinical model to examine the underlying mechanisms and potential interventions for chemotherapy-induced neuropathy. Research by Joseph et al. (2008) demonstrated that cisplatin administration in rodents leads to neuropathic pain-like behavior characterized by mechanical allodynia and thermal hyperalgesia [61]. The study further elucidated the involvement of neuroinflammatory pathways, such as tumor necrosis factor-alpha (TNF-α) signalling, in mediating cisplatin-induced neuropathic pain.

Moreover, preclinical investigations have assessed the efficacy of various pharmaceutical interventions in mitigating the symptoms of CIPN. For instance, studies have explored the neuroprotective effects of natural compounds in animals treated with cisplatin, such as curcumin and resveratrol. These findings have yielded encouraging results, suggesting that these compounds may attenuate cisplatin-induced nerve damage and alleviate neuropathic pain [62,63].

In addition to pharmacological interventions, non-pharmacological approaches have been explored in the CIPN preclinical model. For instance, Currie et al. (2019) showed that physical exercise can improve cisplatin-induced neuropathic pain and nerve function in rodents. This study highlights the potential of exercise therapy as a complementary approach to managing CIPN symptoms [61].

The CIPN preclinical model is a crucial tool for investigating the pathophysiology of chemotherapy-induced peripheral neuropathy and assessing potential therapeutic options. This model accurately reflects the critical features of CIPN observed in human patients, enabling innovative treatments to prevent or alleviate chemotherapy-induced nerve damage. The similarities between DPN and CIPN in preclinical studies offer a valuable foundation for cross-applying findings to improve the understanding and treatment strategies for both conditions. These neuropathies, although originating from distinct causes—diabetes for DPN and chemotherapy for CIPN—display common pathophysiological effects on peripheral nerves, such as oxidative stress, inflammation, and nerve degeneration. The overlap in mechanisms provides an opportunity to use knowledge gained from one model to inform interventions in the other. For example, antioxidant treatments that effectively reduce oxidative damage in CIPN models can be examined in DPN models, potentially leading to broader treatment options for neuropathic conditions. Collaborative research efforts in preclinical settings highlight the potential of combined approaches to enhance the understanding of peripheral neuropathies and develop more effective therapies for these debilitating conditions.

#### 3.1.1. Advantages

One of the primary benefits of this model is its simplicity. By following a specific cisplatin dose regimen, PN can be induced. The ease of induction allows for a wide range of experiments to uncover the intricacies of nerve damage and test potential protective or curative treatments [63].

Furthermore, the model offers flexibility in terms of dosage protocols. Researchers have established various cisplatin administration schedules, ranging from a single dose to multiple doses. These varying protocols enable scientists to explore the impact of cisplatin on both acute and chronic forms of neuropathy, providing a robust framework for examining the efficacy of new therapeutic agents at various stages and severities of the condition [47,64].

This approach aims to not only mitigate the symptoms of neuropathy but also holds potential for preventative strategies that could shield patients from this debilitating side effect, thereby improving their quality of life during and after cancer treatment. By facilitating a detailed study of the pathophysiology of CIPN, this model is a crucial tool in the quest to understand and combat PN in cancer patients.

#### 3.1.2. Disadvantages

While the preclinical model of cisplatin-induced peripheral neuropathy (CIPN) helps study chemotherapy-induced nerve damage, it has several limitations that researchers must consider. One of these limitations is that it does not fully replicate all the clinical features observed in human patients receiving cisplatin chemotherapy. Although CIPN in humans can involve sensory and motor deficits, the preclinical model focuses primarily on sensory symptoms, such as pain and numbness, and may not adequately capture motor impairments [65].

Another drawback is the inconsistency in the initiation and intensity of neuropathy observed in animal models as opposed to human patients. This inconsistency can be attributed to species variations, genetic makeup, and individual susceptibility to cisplatin [66]. Due to these challenges, it is difficult to establish consistent and reproducible neuropathy phenotypes in experimental animals, limiting the reliability and generalizability of study findings.

Furthermore, the CIPN preclinical model may not completely consider the chronic and progressive development of neuropathy in human patients. Although cisplatin treatment in animals has been shown to induce acute neuropathic pain-like behaviors, it is unclear whether the long-term effects of chemotherapy on nerve function and quality of life are adequately captured in short-term experimental studies [67]. The applicability of research findings from animal models to human clinical trials may be restricted by species-specific distinctions in drug metabolism, pharmacokinetics, and neuropathy mechanisms, thereby limiting the potential for direct translation [68]. As a result, therapeutic interventions that show promise in preclinical studies may not always prove effective in treating CIPN in humans.

The severity of cisplatin-induced neurotoxicity is directly related to the cumulative dose administered. Key findings related to dose-dependent toxicity include Low Doses: At lower doses, patients may experience mild and reversible neuropathy characterized by numbness, tingling, and loss of sensation in the extremities. The onset is gradual and may improve after cessation of treatment. Moderate Doses: Intermediate doses result in more pronounced symptoms, including persistent numbness, tingling, and pain. There may be some recovery post-treatment, but residual effects often remain. High Doses: High cumulative doses lead to severe and frequently irreversible neuropathy. Symptoms include severe pain, significant sensory loss, and motor impairment. The onset is rapid, and recovery is limited even after stopping cisplatin [69].

The preclinical model of CIPN offers valuable insights into chemotherapy-induced nerve damage; however, it is essential to recognize and consider its limitations when interpreting study findings and developing therapeutic strategies for clinical translation.

### 3.2. Procedures

Developing a preclinical model of cisplatin-induced peripheral neuropathy (CIPN) involves a series of controlled experiments designed to replicate the neurological effects observed in human patients receiving cisplatin chemotherapy. These experiments usually consist of the following steps:

Animal Selection: The choice of experimental animals, such as rats or mice, is based on age, sex, and strain. Commonly used strains include Sprague-Dawley rats and C57BL/6 mice.

Drug Administration: Cisplatin, a platinum-based chemotherapeutic agent, is administered to animals to induce peripheral neuropathy. The dosage and route of administration may vary depending on the specific experimental protocol. An intraperitoneal injection is a commonly used route for cisplatin administration in preclinical studies.

Dosing Regimen: Cisplatin dosing regimens are established based on eliciting a specific duration and intensity of neuropathy. Multiple cisplatin doses may be administered over several days or weeks to mimic the long-term consequences of chemotherapy treatment.

Behavioral Evaluations: Behavioral tests evaluate the emergence and progression of neuropathic symptoms in the experimental animals. Commonly employed behavioral assays include evaluations of mechanical allodynia, thermal hyperalgesia, and motor deficits (e.g., grip strength, rotarod proficiency).

Histological and Molecular Assessments: Upon completion of the experimental plan, animals may be euthanized and tissues (e.g., dorsal root ganglia, sciatic nerves) harvested for histological and molecular evaluations. These assessments may involve immunohistochemical staining for markers of nerve injury, quantitative polymerase chain reaction (qPCR) for gene expression analysis, and Western blotting for protein quantification.

Data Analysis: The data garnered from behavioral and molecular assessments are subjected to statistical analysis to evaluate the effectiveness of cisplatin in inducing neuropathy and to assess the impact of potential therapeutic interventions.

It is essential to adhere to ethical principles and guidelines for treating and using laboratory animals throughout the experimental procedures to ensure the animals’ welfare.

### 3.3. Collective Studies

Gabapentin and pregabalin are two commonly used anticonvulsants for neuropathic pain; however, studies explicitly focusing on their efficacy in cisplatin-induced neuropathy are limited [70,71,72]. In open-label pilot studies for chemotherapy-induced peripheral neuropathy, platinum-based drugs, such as oxaliplatin and duloxetine, have been explored and have shown promising results in patients with neuropathy [73].

Fluoxetine has garnered interest due to its neuroprotective properties and potential in alleviating CIPN. Its mechanisms include reducing inflammation and oxidative stress and promoting neurogenesis. By affecting serotonin and glutamate, fluoxetine may regulate pain and mitigate neurotoxicity. While promising results have been observed in animal studies, further clinical trials are needed to confirm its efficacy and safety [54].

### 3.4. Mechanisms of Action

Gabapentin, pregabalin, and duloxetine all have mechanisms of action in CINP models that involve modulating neurotransmitter release and improving nerve function, like their roles in diabetic neuropathy, but with specific implications for CINP. Gabapentin works by binding to the α2-δ subunit of voltage-gated calcium channels on central neurons in the context of cisplatin-induced peripheral neuropathy. This interaction reduces the release of excitatory neurotransmitters involved in pain transmission. In CINP models, gabapentin reduces pain symptoms by decreasing neuronal excitability and, thus, the abnormal pain processing associated with nerve damage by cisplatin [74,75].

Pregabalin, much like gabapentin, targets the α2-δ subunit of voltage-dependent calcium channels within the central nervous system. This action results in decreased neurotransmitter release, ultimately attenuating pain signals. In the context of chemotherapy-induced peripheral neuropathy, pregabalin has shown effectiveness in reducing both the frequency and severity of neuropathic pain symptoms while also providing a protective effect on nerve fibres against cisplatin toxicity [72].

Duloxetine, an SNRI, enhances the levels of serotonin and norepinephrine in the nervous system by inhibiting their reuptake at synapses. This increase strengthens pain inhibitory pathways and elevates the pain threshold, making duloxetine particularly beneficial in alleviating painful neuropathic disorders such as CINP. Studies in CINP models indicate that duloxetine can substantially decrease neuropathic pain and enhance the overall quality of life for patients undergoing cisplatin chemotherapy [73].

## 4. Paclitaxel-Induced Peripheral Neuropathy

Paclitaxel-induced peripheral neuropathy (PIPN) is a challenging adverse effect associated with the use of paclitaxel, a chemotherapeutic agent utilized in the treatment of various malignancies, including ovarian, breast, non-small cell lung, and gastric cancers. This type of neuropathy can manifest as numbness, dysesthesia, and pain in the limbs, and it may become a dose-limiting factor in chemotherapy, often necessitating a dose reduction or discontinuation of treatment. However, while there are numerous reports of potential neuropathy inhibitors in preclinical research, very few have shown efficacy in clinical trials. This gap highlights the need for enhanced translational research to bridge the findings from animal models to human clinical settings [76].

Despite the difficulties in finding effective treatments for PIPN, it is worth noting that duloxetine is currently the only recommended treatment according to the American Society of Clinical Oncology’s guidelines. However, more research and development are necessary to establish a broader range of options for patients suffering from this condition [77].

Utilizing a preclinical model of PIPN is highly beneficial for investigating DPN despite the differing etiologies of the two conditions—chemotherapy and diabetes, respectively. This model can be employed to improve understanding and treatment strategies for DPN. Since both PIPN and DPN involve neurobiological mechanisms such as neuronal damage, mitochondrial dysfunction, and increased oxidative stress, examining how paclitaxel induces these mechanisms in PIPN models can provide insights that may also apply to the development and progression of DPN. For example, research in PIPN models demonstrating that paclitaxel results in mitochondrial dysfunction and oxidative stress could aid in identifying comparable pathways in DPN and guide the development of targeted therapies that could alleviate these effects in diabetic patients [78].

Therapeutic interventions, including substances and strategies that have proven effective in mitigating symptoms or halting progression in PIPN models, may be evaluated for their usefulness in DPN models. This approach can accelerate the identification and refinement of treatments for DPN. For example, if specific antioxidants or anti-inflammatory medications reduce neuropathic pain in PIPN models, they could be tested in DPN patients, potentially shortening the time and cost of drug development [79]. Effective pain management strategies are essential to address chronic pain caused by both peripheral ischemic pain in secondary post-occlusive pain and DPN. Novel analgesics and non-pharmacological interventions, which have shown promise in managing pain in PIPN models, may be a helpful starting point for developing effective pain management approaches in DPN. For instance, a regimen of non-opioid pain relievers combined with physical therapy that proves effective in managing PIPN-related pain could be adapted and tested for effectiveness in managing DPN-associated pain [77].

In biomarker discovery, PIPN models are employed to identify biomarkers for peripheral neuropathy, particularly in DPN. Biomarkers are crucial for detecting the condition early, tracking disease progression, and designing personalized treatment plans. For instance, PIPN models can be utilized to investigate serum or tissue biomarkers that indicate the progression of nerve damage in DPN patients, thus facilitating earlier diagnosis and intervention [78]. Translating research findings from PIPN models to DPN requires careful consideration of the differences in etiology between chemotherapy-induced and diabetes-induced neuropathies. To ensure the accurate application of research, studies must carefully evaluate which aspects of PIPN are relevant to DPN. A comprehensive review might examine multiple studies utilizing PIPN models to determine how their results can be applied to DPN, identifying successful translations and areas where PIPN findings do not align with DPN characteristics [80]. The use of PIPN models, which have demonstrated success in assessing chemotherapy-induced neuropathy, holds great potential for advancing research on diabetic neuropathy. Researchers can deepen their knowledge of DPN and improve its clinical management by exploring common pathways, testing therapeutic methods, and evaluating pain management techniques in PIPN models.

### 4.1. Paclitaxel-Induced Peripheral Neuropathy Preclinical Model

Developing a preclinical model to investigate PIPN is essential for understanding its underlying mechanisms and exploring potential therapeutic interventions. Researchers typically employ laboratory animals, such as rodents, in preclinical models of PIPN to simulate the effects of paclitaxel on the peripheral nervous system. This chemotherapy drug disrupts microtubule function in cells, including nerve cells, resulting in nerve damage and dysfunction. By administering paclitaxel to these animals, researchers can replicate the neuropathic symptoms observed in humans undergoing chemotherapy [81]. Additionally, preclinical models allow researchers to evaluate the efficacy and safety of novel treatments for PIPN. This may involve testing pharmacological agents, such as antioxidants, anti-inflammatory drugs, and neuroprotective compounds, that can potentially mitigate paclitaxel-induced nerve damage and alleviate neuropathic symptoms. By assessing these interventions in animal models, researchers can gain valuable insights into their mechanisms of action and determine their potential utility in clinical settings.

Using preclinical models for PIPN is crucial in enhancing our comprehension of this disabling condition and devising effective treatments for cancer patients. Researchers can reproduce significant elements of PIPN in laboratory animals, allowing them to discover novel therapeutic targets, assess potential interventions, and ultimately enhance the quality of life for individuals receiving chemotherapy. Additional research is necessary to create personalized strategies for preventing and managing PIP in clinical practice.

#### 4.1.1. Advantages

The PIPN model, which typically utilizes rodents, replicates the neurotoxic effects of paclitaxel observed in humans, allowing researchers to investigate the underlying mechanisms, potential protective strategies, and therapeutic interventions. The PIPN preclinical model offers several specific advantages, including elucidating mechanistic pathways. The PIPN model aids in understanding the cellular and molecular mechanisms that underlie neuropathy. Studies have demonstrated that paclitaxel affects various cellular components, such as microtubules in dorsal root ganglion neurons, leading to neuropathic pain and neuron dysfunction [82]. The paclitaxel-induced peripheral neuropathy (PIPN) model is beneficial for assessing treatments for peripheral neuropathy without compromising the anticancer activity of paclitaxel [83]. The use of paclitaxel-induced peripheral neuropathy (PIPN) models is crucial for developing and testing new analgesics aimed at relieving chemotherapy-induced neuropathic pain. These models allow detailed investigation of pain pathways and offer valuable insights into the effectiveness of analgesic drugs in managing neuropathic symptoms [83]. An investigation was conducted to determine the efficacy of sigma-1 receptor antagonists in alleviating neuropathic pain in a PIPN model. This study’s findings may contribute to enhancing clinical management strategies for neuropathy in cancer patients. By examining the timing and dosing factors that impact neuropathy development, more refined treatment plans can be devised to minimize adverse effects [84]. In developing predictive biomarkers, research in PIPN models has the potential to contribute to the identification of biomarkers that predict susceptibility or resistance to neuropathy, which can be essential for personalized medicine approaches in cancer therapy [85]. This research establishes genetic indicators suggesting an individual’s likelihood of developing PIPN.

These frameworks offer a regulated environment to methodically explore the consequences of paclitaxel on nerve tissue, providing indispensable insights relevant to clinical contexts and thereby enhancing our comprehension and management of PIPN in cancer patients.

#### 4.1.2. Disadvantages

While preclinical models of PIPN are indispensable for elucidating and devising treatments for neuropathy, they also have certain drawbacks and limitations. These shortcomings can impede the translation of research outcomes from animal models to human clinical settings. The principal disadvantages include: 1. Species and strain variations—the disparities in physiology and genetic composition between humans and the animals employed in PIPN models (commonly rodents) can result in substantial disparities in how neuropathy originates and responds to therapy. This can restrict the relevance of findings to human patients [86]. 2. Paclitaxel-induced peripheral neuropathy (PIPN) models aim to simplify the complex nature of peripheral neuropathy seen in humans. However, these models often fail to account for critical factors such as genetic variability, comorbidities (e.g., diabetes), and prior treatments, which can influence the development of neuropathy and the response to treatment [87]. 3. Variability in dosing and administration—the administration and dosage of paclitaxel in animal models may not necessarily correlate with human clinical treatment regimens, which can consequently impact the development and intensity of neuropathy. As a result, inconsistencies may arise between the observations of neuropathy onset and progression in the animal models and those in patients [87]. 4. Ethical and humane considerations—the incorporation of animals in scientific investigation, particularly in studies that involve the infliction of pain and distress, elicits ethical concerns. While it is imperative to guarantee humane treatment and abide by ethical principles, this simultaneously poses challenges to the planning and implementation of experiments [85]. 5. Financial and time constraints—the establishment and upkeep of animal models for PIPN entail considerable financial and logistical commitments. These costs encompass expenses related to breeding, housing, and providing medical care for the animals and the significant time investment required for long-term research studies [85]. The drawbacks underscore the inherent difficulties and constraints in employing preclinical models to investigate paclitaxel-induced peripheral neuropathy. Although these models are essential for furthering our comprehension and therapy of neuropathy, researchers must critically appraise the extrapolation of their findings to human patients, considering these limitations.

### 4.2. Procedures

Various methodologies are employed to assess PIPN in rodent models, including adjustments to the dose, delivery method, and evaluation techniques. Standard tests for evaluating PIPN are behavioral assays like the Von Frey filaments test for mechanical allodynia, the Hargreaves apparatus for thermal hypo- or hyperalgesia, and electrophysiological tests like nerve conduction studies. Additionally, histologic analyses of nerve tissue can be used to assess axonal degeneration. The choice of species, strain, age, and sex of the animals, as well as the paclitaxel administration regimen, can significantly affect the development of neuropathy and are thus critical variables in the study design [81].

Gabapentin, pregabalin, and duloxetine are often prescribed for the treatment of neuropathic pain conditions, including paclitaxel-induced peripheral neuropathy.

Gabapentin and pregabalin have shown efficacy in reducing pain symptoms in PIPN models [88]. Preclinical studies have shown that gabapentin effectively modulates neuropathic pain [88]. Pregabalin has demonstrated efficacy in reducing neuropathic pain in PIPN through various preclinical studies.

Duloxetine is effective in managing neuropathic pain associated with PIPN by altering the balance of neurotransmitters, thereby decreasing pain perception [89].

PIPN models are crucial for managing chemotherapy-induced neuropathic pain and provide insights into broader applications for other neuropathic conditions.

### 4.3. Collective Studies

Gabapentin and pregabalin are both anticonvulsant medications that have demonstrated effectiveness in alleviating chemotherapy-induced neuropathy in preclinical research and may offer potential benefits in diabetic models as well. For instance, a study may reveal that pregabalin reduces mechanical allodynia and thermal hyperalgesia in a diabetic rat model treated with paclitaxel. This indicates its usefulness in managing neuropathy in diabetic patients undergoing chemotherapy [90].

#### 4.3.1. Duloxetine

Studies in preclinical settings could demonstrate that duloxetine alleviates neuropathic pain symptoms associated with both diabetes and chemotherapy. For example, research might find that duloxetine reduces pain symptoms like mechanical allodynia and thermal hyperalgesia in diabetic models of PIPN, suggesting its effectiveness in dual-pathology scenarios [91].

#### 4.3.2. Combination Therapy

Combination therapy involving gabapentin and duloxetine could offer synergistic effects that are more effective than monotherapy. Research could indicate that using these drugs in combination provides more significant pain relief in diabetic models of PIPN compared to using either drug alone. This approach could be particularly beneficial in patients who have multiple underlying causes of neuropathy, such as those with diabetes undergoing chemotherapy [92].

Investigating these therapeutic options in preclinical diabetic models of PIPN is essential for developing effective treatment strategies that address the complex interplay between chemotherapy-induced and diabetic neuropathies. The promise shown by gabapentin, pregabalin, and duloxetine in preclinical studies supports their further evaluation in clinical trials to confirm their safety and efficacy in managing neuropathic pain in patients with both diabetes and cancer.

## 5. Surgery-Induced or Trauma-Induced Neuropathy

Peripheral neuropathy resulting from surgery or trauma in diabetic patients is a complicated condition exacerbated by the underlying pathology of diabetes. Diabetes mellitus increases the risk of nerve damage due to factors such as hyperglycemia-induced oxidative stress, inflammation, and microvascular dysfunction. Surgery or trauma further complicates the condition by exacerbating nerve injury through mechanical damage, compression, or ischemia, leading to disrupted axonal transport and nerve conduction [93]. The resulting neuropathy often presents as neuropathic pain, characterized by hyperalgesia, allodynia, and spontaneous pain, reflecting sensitization of both peripheral and central nociceptive pathways [94].

The chronic constriction injury (CCI) model is a pioneering method for investigating chronic pain stemming from peripheral nerve damage. Within the spectrum of CCI models, the unilateral loose ligation of the sciatic nerve is favored for its ability to replicate symptoms akin to human peripheral neuropathic pain. This fidelity to human experiences enables researchers to delve into the intricate mechanisms underlying chronic pain and assess the efficacy of therapeutic interventions. Through this model, researchers gain valuable insights into the complexities of chronic pain and pave the way for potential treatment advancements [43].

Therapeutic interventions for peripheral neuropathy include pharmacological agents targeting pain pathways, anti-inflammatory drugs, neuroprotective agents, physical therapy modalities, and surgical interventions to address underlying nerve damage and improve functional outcomes. Despite advances in understanding and treatment, challenges remain in comprehending the complexity of diabetes-related nerve damage and the multifactorial nature of surgical procedures or trauma.

### 5.1. Advantages

Surgery-induced or trauma-induced peripheral neuropathy in diabetic models provides several advantages for investigating the intricacies of diabetic neuropathy and examining potential therapeutic interventions.

Relevance to Clinical Scenario: Diabetic patients undergoing surgery or experiencing trauma worsen their neuropathic complications due to impaired wound healing, altered pain perception, and increased susceptibility to nerve damage. Research on surgery- or trauma-induced peripheral neuropathy in diabetic models closely mirrors the clinical scenario, enhancing the translational relevance of preclinical findings [94]. Surgery or trauma in diabetic patients often leads to exacerbated neuropathic complications due to the pre-existing condition of diabetes, which affects nerve health. By using diabetic models subjected to these stressors, researchers can replicate the clinical scenario more accurately [95]. These models help understand the pathophysiological processes involved in diabetic neuropathy, such as impaired wound healing, altered pain perception, and increased susceptibility to nerve damage [96,97]. This knowledge is crucial for developing targeted therapies. Investigating therapeutic interventions in these models can provide insights into how treatments may perform in actual diabetic patients who experience surgery or trauma. For instance, studies have shown that certain treatments can improve nerve regeneration and reduce neuropathic pain in diabetic models [97]. Using these models, researchers can understand how diabetic neuropathy develops and progresses following injury. For example, studies have shown that diabetic rats subjected to sciatic nerve crush exhibit delayed nerve regeneration and increased pain sensitivity compared to non-diabetic controls, highlighting the impact of diabetes on nerve repair mechanisms [98,99]. Moreover, these models help evaluate the efficacy of potential therapeutic interventions in a setting that closely resembles the clinical challenges faced by diabetic patients.

Controlled Experimental Conditions: Surgical procedures or trauma enable researchers to induce precise and reproducible nerve injuries in diabetic animal models, facilitating the study of specific neuropathic mechanisms and interventions under controlled experimental conditions [100]. In diabetic animal models, controlled induction of nerve injuries through surgical or traumatic means provides a reliable and repeatable approach to studying the pathophysiology of peripheral neuropathy. These methods allow researchers to create consistent injury patterns, which is crucial for understanding the underlying mechanisms of nerve damage and repair in diabetic conditions. For example, nerve crush or transection models in diabetic rodents have been extensively used to investigate the delayed nerve regeneration and heightened pain responses characteristic of diabetic neuropathy [101].

The precision and reproducibility of these experimental models are paramount for testing the efficacy of potential therapeutic interventions. By using controlled injury techniques, researchers can systematically evaluate the impact of various treatments on nerve healing, pain modulation, and functional recovery in diabetic subjects. This approach not only aids in identifying promising therapeutic candidates but also ensures that findings are robust and applicable to clinical settings.

Additionally, controlled experimental conditions enable the isolation of specific variables influencing neuropathy, such as blood glucose levels, oxidative stress, and inflammatory responses. This isolation is critical for dissecting the multifaceted nature of diabetic neuropathy and developing targeted interventions that address the distinct pathological features observed in diabetic patients [102].

Evaluation of Therapeutic Interventions: Surgery- or trauma-induced peripheral neuropathy models allow researchers to assess the efficacy of various therapeutic interventions, including pharmacological agents, neuroprotective strategies, and surgical approaches, in mitigating neuropathic pain and promoting nerve regeneration [94]. Peripheral neuropathy, particularly those involving surgically or traumatically induced nerve injuries, is crucial for evaluating the potential benefits of therapeutic interventions. These models offer a structured setting to rigorously and systematically explore the impacts of various treatments. For example, pharmacological agents such as gabapentin, pregabalin, and duloxetine have been tested in these models to determine their efficacy in reducing neuropathic pain and improving nerve function [103].

Neuroprotective strategies, including the use of antioxidants, growth factors, and anti-inflammatory agents, have also been explored in surgery- or trauma-induced diabetic neuropathy models. These interventions aim to protect nerve cells from damage, enhance nerve regeneration, and reduce pain. For instance, the application of nerve growth factor (NGF) has shown promising results in promoting nerve regeneration and functional recovery in diabetic neuropathy models [104].

Furthermore, surgical approaches such as nerve grafting and decompression surgeries have been evaluated for their ability to restore nerve function and alleviate pain in diabetic neuropathy. Studies have demonstrated that these surgical interventions can improve outcomes by providing a conducive environment for nerve repair and reducing mechanical compression on nerves [105,106].

Surgery- or trauma-induced peripheral neuropathy models provide valuable insights into the pathophysiological mechanisms underlying diabetic neuropathy, including oxidative stress, inflammation, neurovascular dysfunction, and impaired nerve regeneration, facilitating the development of targeted therapeutic strategies [95,96,97].

Diabetic neuropathy is a complex condition characterized by multiple pathophysiological processes that contribute to nerve damage. Using surgery- or trauma-induced peripheral neuropathy models, researchers can dissect these intricate mechanisms in a controlled setting, providing a deeper understanding of the disease. Oxidative stress plays a crucial role in the development and progression of diabetic neuropathy. Elevated blood glucose levels in diabetes lead to the generation of reactive oxygen species (ROS), which cause significant damage to nerve cells. Animal models have demonstrated that oxidative stress markers are elevated in diabetic nerves, and interventions targeting oxidative stress can ameliorate neuropathic symptoms [107,108]. Chronic inflammation is another key contributor to diabetic neuropathy. Inflammatory cytokines and immune cells infiltrate nerve tissues, exacerbating nerve damage and pain. Studies using diabetic models have shown that anti-inflammatory treatments can reduce nerve inflammation and improve nerve function [109]. Neurovascular dysfunction, including reduced blood flow and impaired endothelial function, is commonly observed in diabetic neuropathy. This dysfunction leads to decreased oxygen and nutrient supply to nerves, further contributing to nerve degeneration. Research using animal models has highlighted the importance of vascular health in maintaining nerve integrity and has identified potential therapeutic targets to improve neurovascular function [110,111]. Diabetes impairs the natural regenerative capacity of nerves, delaying recovery from injury. Diabetic models have shown that nerve growth factors and other regenerative pathways are dysregulated in diabetes, leading to poor nerve repair. Investigating these pathways in controlled models has facilitated the development of strategies to enhance nerve regeneration in diabetic patients [112,113,114].

Investigation of nerve regeneration enables researchers to investigate nerve regeneration processes following surgical or traumatic injury in diabetic conditions, elucidating the factors influencing axonal growth, myelination, and functional recovery [96]. These models enable researchers to investigate nerve regeneration processes following surgical or traumatic injury in diabetic conditions, elucidating the factors influencing axonal growth, myelination, and functional recovery.

Diabetes significantly impairs the ability of nerves to regenerate after injury, posing a major challenge for the effective treatment of diabetic neuropathy. By using surgery- or trauma-induced peripheral neuropathy models in diabetic conditions, researchers can closely examine the underlying mechanisms that affect nerve repair and regeneration. Axonal regeneration is crucial for the recovery of nerve function following injury. In diabetic conditions, high glucose levels and associated metabolic disturbances inhibit axonal growth. Research using diabetic models has shown that hyperglycemia impairs the expression of key growth factors and signalling pathways involved in axonal regeneration [115]. For instance, the downregulation of neurotrophic factors such as nerve growth factor (NGF) and brain-derived neurotrophic factor (BDNF) in diabetic nerves leads to reduced axonal outgrowth and regeneration. The process of forming the protective myelin sheath around axons is critical for efficient nerve signal transmission and functional recovery. Diabetic neuropathy models have demonstrated that diabetes disrupts myelination processes, resulting in thinner and dysfunctional myelin sheaths [116]. This demyelination contributes to the sensory and motor deficits observed in diabetic patients. Studies have highlighted the role of Schwann cells, the myelinating cells of the peripheral nervous system, in mediating myelin repair and the impact of diabetes on their function. Functional recovery after nerve injury depends on the successful regeneration of axons and the restoration of proper myelination. Diabetic models have been instrumental in identifying factors that promote or hinder functional recovery. For example, research has shown that oxidative stress and chronic inflammation in diabetic conditions impede the functional recovery of nerves by exacerbating nerve damage and inhibiting regenerative processes [117,118]. Additionally, therapeutic interventions targeting these factors have enhanced functional outcomes in diabetic neuropathy models.

Researchers can gain valuable insights into the pathophysiology of diabetic neuropathy and advance the development of novel therapeutic approaches for this debilitating condition by utilizing surgery- or trauma-induced peripheral neuropathy models in diabetic animals.

### 5.2. Disadvantages

Peripheral neuropathy resulting from surgery or trauma in diabetic models presents both valuable insights and several drawbacks and obstacles.

Surgical procedures or trauma-induced nerve injuries introduce complexity and variability in experimental outcomes due to variations in the extent and nature of nerve damage, wound healing processes, and individual differences in animals [119].

Surgery or trauma-induced peripheral neuropathy models involve invasive procedures that may cause additional tissue damage, inflammation, and stress responses in animals, potentially confounding experimental results and compromising animal welfare [119].

Standardizing surgical procedures or trauma-induced nerve injuries across experiments and different research groups poses challenges, leading to variability in the severity and outcomes of neuropathy models, which may affect the reproducibility and reliability of study findings [119]. Standardizing surgical procedures or trauma-induced nerve injuries across experiments and different research groups poses challenges, leading to variability in the severity and outcomes of neuropathy models, which may affect the reproducibility and reliability of study findings. One of the significant challenges in using surgery- or trauma-induced neuropathy models is the difficulty in achieving consistent and reproducible results across different studies. Variability in surgical techniques, the extent of nerve damage, and post-operative care can lead to differences in the severity of neuropathy and the outcomes observed in experiments [120]. Precisely executing surgical procedures is critical for inducing consistent nerve injuries. Even minor deviations in the technique can result in varying degrees of nerve damage, affecting the study outcomes. For example, the amount of pressure applied during a nerve crush injury, or the exact location and extent of a nerve transection can significantly influence the severity of neuropathy [120]. Standardizing these procedures across different laboratories is challenging and requires meticulous training and detailed protocols to ensure uniformity. Differences in post-operative care, which includes pain management, infection control, and overall animal health monitoring, can also influence variability in study outcomes. Differences in housing conditions, pain management strategies, and monitoring of diabetic status can affect the recovery and behavior of the animals, leading to inconsistencies in the results [121,122]. Standardized protocols for post-operative care are essential to minimize these variables and improve the reproducibility of findings. The lack of standardization can undermine the reproducibility and reliability of neuropathy studies. Inconsistent results make it difficult to compare findings across different research groups and to draw definitive conclusions about the efficacy of therapeutic interventions. This variability can also complicate translating preclinical findings to clinical settings, as inconsistent preclinical data may not accurately reflect the potential benefits or risks of treatments in human patients [123].

While surgery-induced or trauma-induced peripheral neuropathy models may replicate aspects of diabetic neuropathy, they may not fully capture the chronic, progressive nature of the condition and its associated metabolic, vascular, and systemic abnormalities, limiting their clinical relevance [124,125].

The invasive nature of surgical procedures or trauma-induced nerve injuries raises ethical concerns regarding animal welfare, pain management, and the justification of inducing neuropathy in animals, necessitating adherence to ethical guidelines and regulations [126].

Conducting surgical procedures or trauma-induced nerve injuries in diabetic models requires specialized equipment, expertise, and resources, making them costly and resource-intensive, particularly for large-scale or longitudinal studies.

Although there are limitations to surgery-induced or trauma-induced peripheral neuropathy in diabetic models, it is still a valuable tool for studying certain aspects of diabetic neuropathy and investigating potential therapeutic interventions.

### 5.3. Procedures

**Figure 8:**
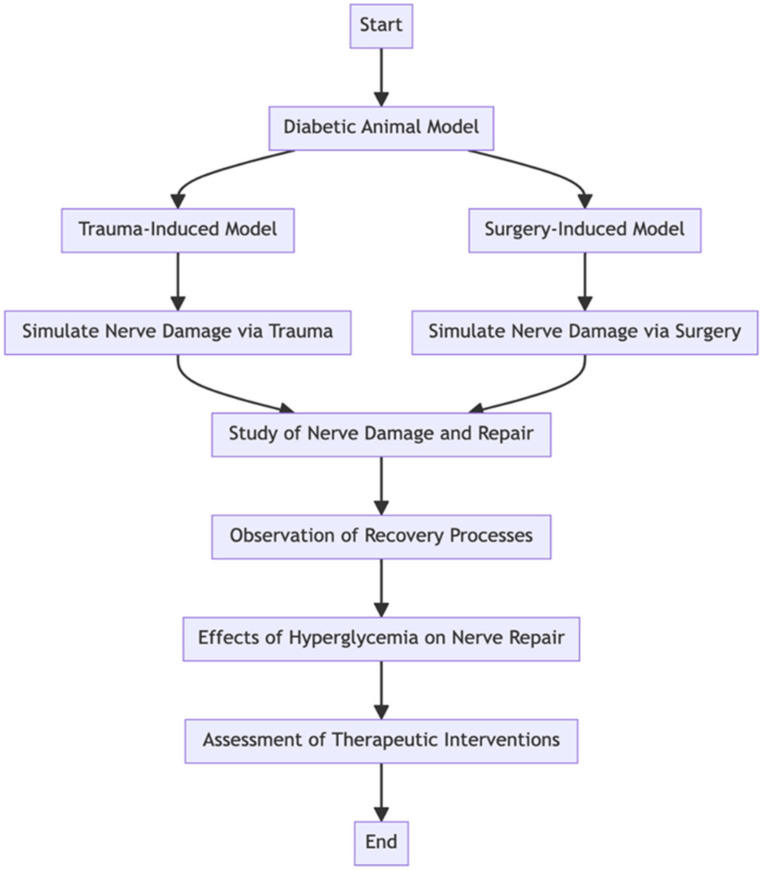
A diagram demonstrating the procedures and methodologies associated with the utilization of surgery-induced and trauma-induced models of peripheral neuropathy in diabetic animals.

Surgery-induced or trauma-induced peripheral neuropathy models in diabetic animals involve precise surgical procedures or traumatic injury to induce nerve damage, mimicking aspects of diabetic neuropathy [96]. These procedures typically entail exposing and manipulating peripheral nerves under anesthesia, which may include nerve transection, ligation, compression, or crush (Figure 8). The choice of nerve for manipulation or injury depends on the research objectives and the specific neuropathic pain condition being studied, with commonly targeted nerves including the sciatic or saphenous nerve [127].

In diabetic models, peripheral neuropathy can be induced alongside surgical or traumatic nerve injury through methods such as streptozotocin injection or genetic manipulation to cause hyperglycemia. Following the procedure, the development and progression of peripheral neuropathy in diabetic animals are evaluated using various behavioral, electrophysiological, histological, and biochemical methods. These assessments measure sensory deficits, nerve conduction abnormalities, nerve morphology, and molecular changes associated with neuropathic pain [128].

While these models allow researchers to study the pathophysiology and therapeutic interventions for diabetic neuropathy in a controlled experimental setting, they also present challenges such as complexity, variability, and ethical considerations regarding animal welfare and pain management. However, despite these limitations, surgery- or trauma-induced peripheral neuropathy models remain valuable tools for advancing our understanding and treatment of diabetic neuropathy [128].

### 5.4. Other Surgical Models

Spared Nerve Injury (SNI) Model: The SNI model involves ligating and severing the tibial and common peroneal nerves while leaving the sural nerve intact. This model helps study the development of neuropathic pain and testing new treatments for nerve injury-related pain [129].

Partial Sciatic Nerve Ligation (PSNL) Model: In the PSNL model, a partial ligation of the sciatic nerve is performed, causing consistent and reproducible neuropathic pain. This model is precious for investigating the pathophysiology of chronic neuropathic pain and evaluating therapeutic interventions [130].

Nerve Crush Injury Model: The nerve crush injury model involves applying controlled pressure to the sciatic nerve to induce axonal damage without severing the nerve. This model is often used to study nerve regeneration and the effects of potential neuroprotective agents [98].

Relevance to Neuropathy Conditions: These surgical models are not limited to diabetic neuropathy but are also employed in studying various forms of peripheral neuropathy, including those induced by chemotherapy (e.g., CIPN) and traumatic injuries. Incorporating multiple models provides a broader understanding of neuropathic mechanisms and enhances the translational potential of preclinical findings [130,131,132].

### 5.5. Collective Studies

Several studies have explored the effectiveness of gabapentin, pregabalin, and duloxetine in managing neuropathic pain in diabetic animals with surgery-induced or trauma-induced peripheral neuropathy. These investigations aim to elucidate the potential therapeutic benefits of these drugs and their mechanisms of action in diabetic neuropathy.

Regarding gabapentin, research has demonstrated its efficacy in alleviating neuropathic pain in surgery-induced or trauma-induced peripheral neuropathy models in diabetic animals. Studies suggest that gabapentin reduces pain behavior and improves nerve function by modulating calcium channel activity and inhibiting excitatory neurotransmitter release [133].

Similarly, collective studies have shown that pregabalin effectively attenuates neuropathic pain in surgery-induced or trauma-induced peripheral neuropathy models in diabetic animals. Pregabalin exerts analgesic effects and reduces neurotransmitter release and neuronal excitability [38].

Duloxetine has demonstrated its effectiveness in alleviating neuropathic pain in animal models of surgery-induced or trauma-induced peripheral neuropathy in diabetic patients. As a serotonin–noradrenaline reuptake inhibitor, duloxetine modifies descending pain pathways and suppresses pain transmission, enhancing pain relief and improving functional outcomes [91].

Together, studies on gabapentin, pregabalin, and duloxetine emphasize their therapeutic potential for treating neuropathic pain in surgery-induced or trauma-induced peripheral neuropathy models in diabetic animals. These studies provide valuable insights into these medications’ mechanisms of action and efficacy, which can contribute to developing new treatments for diabetic neuropathy.

All preclinical methods for screening peripheral neuropathies have been compared for easy understanding (Table 2).

## 6. Conclusions

Preclinical models are essential in advancing knowledge about PN and developing effective treatments. Models such as the STZ-induced diabetic rat, cisplatin-induced, paclitaxel-induced, and trauma-induced models have contributed valuable information regarding disease mechanisms and potential therapeutic agents. Despite their limitations, these models have played a crucial role in the progress made in the field. Future research should focus on improving these models to reflect better human PN and exploring combinatorial or novel models that account for the multifaceted nature of this condition. Such advancements are necessary for the discovery of more effective treatments for PN.

## Figures and Tables

**Table 2:** Comparison and contrast of preclinical models used to study peripheral neuropathy in diabetic models, including disease-induced, cisplatin-induced, paclitaxel-induced, and surgery-induced models.

Aspect	Disease-Induced Model	Cisplatin-Induced Model	Paclitaxel-Induced Model	Surgery-Induced Model
**Induction Method**	Chemically induced (e.g., streptozotocin injection)	Administration of cisplatin	Administration of paclitaxel	Surgical intervention (e.g., nerve ligation)
**Underlying Mechanism**	High blood sugar levels leading to nerve damage	Direct DNA damage and oxidative stress	Microtubule stabilisation leading to nerve damage	Mechanical injury to nerves, leading to neuropathy
**Relevance to Peripheral Neuropathy**	Peripheral neuropathy shares similarities with diabetic neuropathy	Chemotherapy-induced peripheral neuropathy	Chemotherapy-induced peripheral neuropathy	It may mimic diabetic neuropathy or exacerbate it
**Symptomatology**	Neuropathic pain, numbness, tingling, weakness	Neuropathic pain, numbness, tingling, weakness	Neuropathic pain, numbness, tingling, weakness	Neuropathic pain, numbness, tingling, weakness
**Experimental Control**	Moderate	High	High	Moderate to high
**Therapeutic Studies**	Ghasemi et al. (2023) [20]	Gu et al. (2021) [134]	Staff et al. (2020) [135]	Attal et al. (2018) [136]

Disease-induced model—STZ–induced PN model, cisplatin-induced model—CIPN model, paclitaxel-induced model—PIPN model, surgery-induced model—SIPN model.
